# (*E*)-1-(4-Benzhydrylpiperazin-1-yl)-3-(3,4-dieth­oxy­phen­yl)prop-2-en-1-one ethanol monosolvate

**DOI:** 10.1107/S1600536811042267

**Published:** 2011-10-22

**Authors:** Yan Zhong, Bin Wu

**Affiliations:** a School of Chemistry and Chemical Engineering, Southeast University, Sipailou No. 2 Nanjing, Nanjing 210096, People’s Republic of China; b School of Pharmacy, Nanjing Medical University, Hanzhong Road No. 140 Nanjing, Nanjing 210029, People’s Republic of China

## Abstract

In the title compound, C_30_H_34_N_2_O_3_·C_2_H_6_O, the piperazine ring adopts a chair conformation and the ethene bond exhibits an *E* conformation. In the crystal, the two components are linked by an O—H⋯O hydrogen bond.

## Related literature

For biological properties of cinnamic acid derivatives, see: Shi *et al.* (2005 ▶); Qian *et al.* (2010 ▶). For the synthesis, see: Wu *et al.* (2008 ▶). For a related structure, see: Teng *et al.* (2011 ▶).
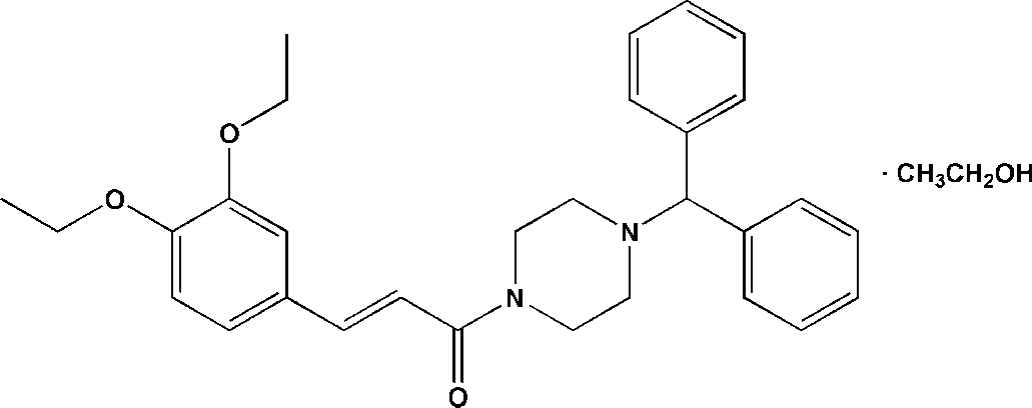


## Experimental

### 

#### Crystal data

C_30_H_34_N_2_O_3_·C_2_H_6_O
*M* *_r_* = 516.66
Triclinic, 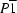

*a* = 7.9590 (16) Å
*b* = 12.039 (2) Å
*c* = 16.298 (3) Å
α = 104.27 (3)°
β = 100.09 (3)°
γ = 100.02 (3)°
*V* = 1450.9 (5) Å^3^
*Z* = 2
Mo *K*α radiation
μ = 0.08 mm^−1^
*T* = 293 K
0.30 × 0.20 × 0.10 mm

#### Data collection

Enraf–Nonius CAD-4 diffractometer
Absorption correction: ψ scan (North *et al.* 1968 ▶) *T*_min_ = 0.977, *T*_max_ = 0.992
5753 measured reflections
5335 independent reflections
3341 reflections with *I* > 2.0σ(*I*)
*R*_int_ = 0.019
3 standard reflections every 200 reflections  intensity decay: 1%

#### Refinement

*R*[*F* ^2^ > 2σ(*F* ^2^)] = 0.067
*wR*(*F* ^2^) = 0.166
*S* = 1.00
5335 reflections
343 parameters
1 restraint
H-atom parameters constrained
Δρ_max_ = 0.34 e Å^−3^
Δρ_min_ = −0.25 e Å^−3^

### 

Data collection: *CAD-4 EXPRESS* (Enraf–Nonius, 1994 ▶); cell refinement: *CAD-4 EXPRESS*; data reduction: *XCAD4* (Harms & Wocadlo, 1995 ▶); program(s) used to solve structure: *SHELXS97* (Sheldrick, 2008 ▶); program(s) used to refine structure: *SHELXL97* (Sheldrick, 2008 ▶); molecular graphics: *SHELXL97* (Sheldrick, 2008 ▶); software used to prepare material for publication: *SHELXL97*.

## Supplementary Material

Crystal structure: contains datablock(s) I, global. DOI: 10.1107/S1600536811042267/pv2451sup1.cif

Structure factors: contains datablock(s) I. DOI: 10.1107/S1600536811042267/pv2451Isup2.hkl

Supplementary material file. DOI: 10.1107/S1600536811042267/pv2451Isup3.cml

Additional supplementary materials:  crystallographic information; 3D view; checkCIF report

## Figures and Tables

**Table 1:** Hydrogen-bond geometry (Å, °)

*D*—H⋯*A*	*D*—H	H⋯*A*	*D*⋯*A*	*D*—H⋯*A*
O4—H4*B*⋯O3	0.82	1.94	2.765 (3)	177

**Fig. 1.**
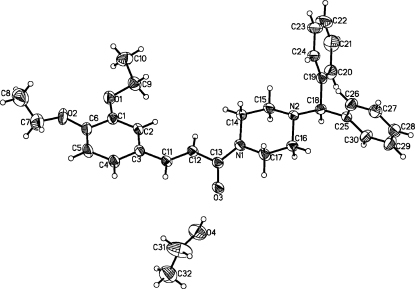
The molecular structure and numbering scheme of the title compound. Displacement ellipsoids for non-H atoms are drawn at the 50% probability level.

**Fig. 2.**
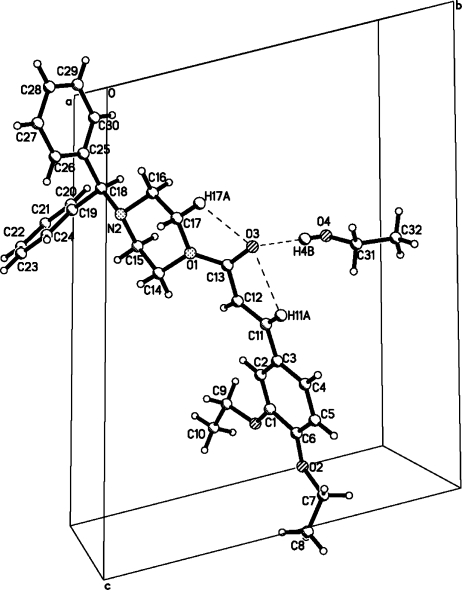
A view of the unit cell of the title compound showing hydrogen bonds.

## supplementary crystallographic information

### Comment

There has been much research interest in cinnamic acid and its derivatives due to their biological activities (Shi *et al.*, 2005; Qian *et al.*, 2010). In this work, we report the crystal structure of the title compound. The title compound (Fig. 1) exists an E-conformation with respect to the C11═C12 ethene bond [1.327 (3) Å] and the torsion angle C3—C11—C12—C13 is 175.4 (2)°. The piperazine ring adopts a chair conformation. The molecular structure is stabilized by intramolecular O—H···O and C—H···O interactions between the title compound and the ethanol molecule of solvation.

### Experimental

The synthesis follows the method of Wu *et al.* (2008). The title compound was prepared by stirring a mixture of (*E*)-3-(3,4-diethoxyphenyl)acrylic acid (0.945 g; 4 mmol), thionyl chloride (2 ml) and dichloromethane (30 ml) for 6 h at room temperature. The solvent was removed under reduced pressure. The residue was dissolved in acetone (15 ml) and reacted with 1-benzhydrylpiperazine (1.514 g; 6 mmol) in the presence of triethylamine (5 ml) for 12 h at room temperature. The resultant mixture was cooled. The solid, (*E*)-1-(4-(benzhydrylpiperazin-1-yl)-3-(3,4-diethoxyphenyl)prop-2-en-1- one obtained was filtered and was recrystallized from ethanol. The pale-yellow single crystals of the title compound used in *X*-ray diffraction studies were grown in ethanol by slow evaporation at room temperature.

### Refinement

All hydrogen atoms were positioned geometrically with C—H distances ranging from 0.93 to 0.98 Å and refined as riding on their parent atoms with *U*ĩso~(H) = 1.2 or 1.5*U*~eq~ of the carrier atoms.

### Crystal data

**Table:**

C_30_H_34_N_2_O_3_·C_2_H_6_O	*Z* = 2
*M**_r_* = 516.66	*F*(000) = 556
Triclinic, *P*1	*D*_x_ = 1.183 Mg m^−^^3^
Hall symbol: -P 1	Mo *K*α radiation, λ = 0.71073 Å
*a* = 7.9590 (16) Å	Cell parameters from 25 reflections
*b* = 12.039 (2) Å	θ = 10–13°
*c* = 16.298 (3) Å	µ = 0.08 mm^−^^1^
α = 104.27 (3)°	*T* = 293 K
β = 100.09 (3)°	Block, pale-yellow
γ = 100.02 (3)°	0.30 × 0.20 × 0.10 mm
*V* = 1450.9 (5) Å^3^	

### Data collection

**Table:**

Enraf–Nonius CAD-4 diffractometer	3341 reflections with *I* > 2.0σ(*I*)
Radiation source: fine-focus sealed tube	*R*_int_ = 0.019
graphite	θ_max_ = 25.4°, θ_min_ = 1.3°
ω/2θ scans	*h* = 0→9
Absorption correction: ψ scan (North *et al.* 1968)	*k* = −14→14
*T*_min_ = 0.977, *T*_max_ = 0.992	*l* = −19→19
5753 measured reflections	3 standard reflections every 200 reflections
5335 independent reflections	intensity decay: 1%

### Refinement

**Table:**

Refinement on *F*^2^	Primary atom site location: structure-invariant direct methods
Least-squares matrix: full	Secondary atom site location: difference Fourier map
*R*[*F*^2^ > 2σ(*F*^2^)] = 0.067	Hydrogen site location: inferred from neighbouring sites
*wR*(*F*^2^) = 0.166	H-atom parameters constrained
*S* = 1.00	*w* = 1/[σ^2^(*F*_o_^2^) + (0.060*P*)^2^ + 0.870*P*] where *P* = (*F*_o_^2^ + 2*F*_c_^2^)/3
5335 reflections	(Δ/σ)_max_ < 0.001
343 parameters	Δρ_max_ = 0.34 e Å^−^^3^
1 restraint	Δρ_min_ = −0.25 e Å^−^^3^

### Special details

**Table:**

Geometry. All e.s.d.'s (except the e.s.d. in the dihedral angle between two l.s. planes) are estimated using the full covariance matrix. The cell e.s.d.'s are taken into account individually in the estimation of e.s.d.'s in distances, angles and torsion angles; correlations between e.s.d.'s in cell parameters are only used when they are defined by crystal symmetry. An approximate (isotropic) treatment of cell e.s.d.'s is used for estimating e.s.d.'s involving l.s. planes.
Refinement. Refinement of *F*^2^ against ALL reflections. The weighted *R*-factor *wR* and goodness of fit *S* are based on *F*^2^, conventional *R*-factors *R* are based on *F*, with *F* set to zero for negative *F*^2^. The threshold expression of *F*^2^ > σ(*F*^2^) is used only for calculating *R*-factors(gt) *etc*. and is not relevant to the choice of reflections for refinement. *R*-factors based on *F*^2^ are statistically about twice as large as those based on *F*, and *R*- factors based on ALL data will be even larger.

### Fractional atomic coordinates and isotropic or equivalent isotropic displacement parameters (Å^2^)

**Table:**

	*x*	*y*	*z*	*U*_iso_*/*U*_eq_	
N1	0.2329 (3)	0.27311 (19)	0.89941 (14)	0.0549 (6)	
O1	0.7626 (3)	0.55168 (18)	1.34911 (12)	0.0661 (6)	
C1	0.6100 (4)	0.5709 (2)	1.30918 (16)	0.0476 (6)	
N2	0.3066 (3)	0.07607 (17)	0.78242 (12)	0.0430 (5)	
C2	0.5375 (3)	0.5301 (2)	1.22233 (16)	0.0452 (6)	
H2A	0.5948	0.4863	1.1858	0.054*	
O2	0.6018 (3)	0.6682 (2)	1.45078 (13)	0.0867 (7)	
O3	0.1351 (2)	0.43931 (16)	0.91346 (12)	0.0595 (5)	
C3	0.3771 (3)	0.5530 (2)	1.18677 (16)	0.0451 (6)	
C4	0.2969 (4)	0.6201 (2)	1.24238 (18)	0.0543 (7)	
H4A	0.1924	0.6379	1.2199	0.065*	
C5	0.3690 (4)	0.6618 (3)	1.33157 (19)	0.0625 (8)	
H5A	0.3130	0.7073	1.3678	0.075*	
C6	0.5231 (4)	0.6356 (2)	1.36630 (17)	0.0552 (7)	
C7	0.5048 (6)	0.7119 (4)	1.5114 (3)	0.1090 (14)	
H7A	0.5415	0.7971	1.5312	0.131*	
H7B	0.3816	0.6913	1.4825	0.131*	
C8	0.5255 (8)	0.6681 (4)	1.5834 (3)	0.1344 (19)	
H8A	0.4611	0.7032	1.6234	0.202*	
H8B	0.6475	0.6868	1.6117	0.202*	
H8C	0.4822	0.5842	1.5647	0.202*	
C9	0.8473 (4)	0.4750 (3)	1.2980 (2)	0.0685 (8)	
H9A	0.8909	0.5102	1.2561	0.082*	
H9B	0.7658	0.4005	1.2667	0.082*	
C10	0.9962 (5)	0.4564 (3)	1.3597 (2)	0.0879 (11)	
H10A	1.0571	0.4052	1.3277	0.132*	
H10B	0.9512	0.4214	1.4007	0.132*	
H10C	1.0756	0.5308	1.3903	0.132*	
C11	0.2904 (3)	0.5049 (2)	1.09455 (16)	0.0462 (6)	
H11A	0.1912	0.5312	1.0762	0.055*	
C12	0.3369 (3)	0.4281 (2)	1.03407 (16)	0.0443 (6)	
H12A	0.4399	0.4036	1.0484	0.053*	
C13	0.2287 (3)	0.3812 (2)	0.94486 (16)	0.0459 (6)	
C14	0.3237 (4)	0.1914 (2)	0.93083 (17)	0.0608 (8)	
H14A	0.2394	0.1285	0.9387	0.073*	
H14B	0.4038	0.2321	0.9866	0.073*	
C15	0.4242 (4)	0.1404 (2)	0.86614 (16)	0.0547 (7)	
H15A	0.5092	0.2035	0.8589	0.066*	
H15B	0.4875	0.0879	0.8881	0.066*	
C16	0.2163 (4)	0.1587 (2)	0.75049 (17)	0.0550 (7)	
H16A	0.1342	0.1167	0.6956	0.066*	
H16B	0.3016	0.2185	0.7399	0.066*	
C17	0.1189 (4)	0.2173 (3)	0.81343 (18)	0.0613 (8)	
H17A	0.0708	0.2762	0.7919	0.074*	
H17B	0.0221	0.1592	0.8175	0.074*	
C18	0.4062 (3)	0.0275 (2)	0.71917 (16)	0.0446 (6)	
H18A	0.4866	0.0939	0.7116	0.054*	
C19	0.5164 (3)	−0.0494 (2)	0.75393 (16)	0.0469 (6)	
C20	0.6872 (4)	−0.0369 (3)	0.7467 (2)	0.0681 (8)	
H20A	0.7347	0.0180	0.7208	0.082*	
C21	0.7907 (5)	−0.1079 (4)	0.7786 (3)	0.0954 (13)	
H21A	0.9071	−0.0987	0.7744	0.114*	
C22	0.7220 (6)	−0.1890 (4)	0.8153 (3)	0.0970 (13)	
H22A	0.7902	−0.2366	0.8351	0.116*	
C23	0.5508 (5)	−0.2011 (3)	0.8234 (2)	0.0787 (10)	
H23A	0.5036	−0.2561	0.8494	0.094*	
C24	0.4503 (4)	−0.1317 (2)	0.79291 (18)	0.0560 (7)	
H24A	0.3349	−0.1403	0.7986	0.067*	
C25	0.2854 (3)	−0.0390 (2)	0.63059 (16)	0.0465 (6)	
C26	0.1525 (4)	−0.1347 (3)	0.61976 (19)	0.0612 (8)	
H26A	0.1337	−0.1593	0.6679	0.073*	
C27	0.0458 (4)	−0.1954 (3)	0.5384 (2)	0.0714 (9)	
H27A	−0.0433	−0.2600	0.5324	0.086*	
C28	0.0710 (5)	−0.1608 (3)	0.4677 (2)	0.0783 (10)	
H28A	0.0001	−0.2017	0.4131	0.094*	
C29	0.2002 (5)	−0.0662 (4)	0.4774 (2)	0.0816 (10)	
H29A	0.2164	−0.0414	0.4291	0.098*	
C30	0.3098 (4)	−0.0052 (3)	0.55870 (18)	0.0649 (8)	
H30A	0.3997	0.0587	0.5639	0.078*	
O4	0.2562 (4)	0.6771 (2)	0.93685 (19)	0.1012 (8)	
H4B	0.2169	0.6069	0.9299	0.152*	
C31	0.1344 (6)	0.7409 (4)	0.9627 (4)	0.141 (2)	
H31A	0.1154	0.7288	1.0173	0.169*	
H31B	0.0242	0.7071	0.9200	0.169*	
C32	0.1745 (6)	0.8642 (4)	0.9737 (3)	0.1165 (15)	
H32A	0.0788	0.8966	0.9896	0.175*	
H32B	0.1930	0.8786	0.9202	0.175*	
H32C	0.2788	0.9007	1.0187	0.175*	

### Atomic displacement parameters (Å^2^)

**Table:**

	*U*^11^	*U*^22^	*U*^33^	*U*^12^	*U*^13^	*U*^23^
N1	0.0656 (15)	0.0441 (12)	0.0470 (13)	0.0229 (11)	−0.0013 (11)	0.0016 (10)
O1	0.0691 (13)	0.0717 (13)	0.0511 (11)	0.0270 (11)	0.0043 (10)	0.0053 (10)
C1	0.0533 (16)	0.0424 (14)	0.0451 (15)	0.0079 (12)	0.0130 (12)	0.0099 (12)
N2	0.0505 (12)	0.0373 (11)	0.0388 (11)	0.0160 (9)	0.0029 (9)	0.0076 (9)
C2	0.0495 (15)	0.0387 (13)	0.0467 (15)	0.0124 (12)	0.0172 (12)	0.0049 (11)
O2	0.1123 (19)	0.1058 (18)	0.0404 (12)	0.0440 (15)	0.0217 (12)	0.0016 (12)
O3	0.0652 (12)	0.0551 (11)	0.0558 (11)	0.0305 (10)	0.0028 (9)	0.0076 (9)
C3	0.0500 (15)	0.0390 (13)	0.0453 (14)	0.0115 (12)	0.0138 (12)	0.0071 (11)
C4	0.0537 (16)	0.0562 (16)	0.0541 (17)	0.0203 (14)	0.0171 (13)	0.0087 (13)
C5	0.074 (2)	0.0623 (18)	0.0578 (18)	0.0273 (16)	0.0328 (16)	0.0085 (15)
C6	0.0685 (19)	0.0502 (16)	0.0464 (16)	0.0119 (14)	0.0217 (14)	0.0079 (13)
C7	0.137 (4)	0.132 (4)	0.077 (3)	0.072 (3)	0.039 (3)	0.025 (3)
C8	0.197 (6)	0.113 (4)	0.131 (4)	0.056 (4)	0.095 (4)	0.047 (3)
C9	0.065 (2)	0.068 (2)	0.070 (2)	0.0210 (16)	0.0131 (16)	0.0118 (16)
C10	0.076 (2)	0.101 (3)	0.090 (3)	0.031 (2)	0.002 (2)	0.036 (2)
C11	0.0463 (15)	0.0433 (14)	0.0509 (15)	0.0171 (12)	0.0129 (12)	0.0110 (12)
C12	0.0429 (14)	0.0408 (13)	0.0484 (15)	0.0140 (11)	0.0088 (12)	0.0093 (11)
C13	0.0427 (14)	0.0467 (15)	0.0489 (15)	0.0159 (12)	0.0100 (12)	0.0112 (12)
C14	0.089 (2)	0.0506 (16)	0.0404 (15)	0.0298 (16)	0.0029 (14)	0.0064 (12)
C15	0.0650 (18)	0.0449 (15)	0.0477 (15)	0.0237 (14)	−0.0051 (13)	0.0060 (12)
C16	0.0648 (18)	0.0475 (15)	0.0471 (15)	0.0218 (14)	−0.0038 (13)	0.0084 (12)
C17	0.0624 (18)	0.0538 (17)	0.0545 (17)	0.0241 (14)	−0.0064 (14)	−0.0021 (13)
C18	0.0481 (15)	0.0430 (14)	0.0439 (14)	0.0099 (12)	0.0089 (12)	0.0162 (11)
C19	0.0465 (15)	0.0443 (14)	0.0441 (14)	0.0122 (12)	0.0037 (12)	0.0053 (12)
C20	0.0583 (19)	0.081 (2)	0.0639 (19)	0.0196 (17)	0.0152 (15)	0.0164 (17)
C21	0.062 (2)	0.133 (4)	0.093 (3)	0.051 (2)	0.009 (2)	0.023 (3)
C22	0.102 (3)	0.087 (3)	0.104 (3)	0.057 (3)	−0.005 (2)	0.026 (2)
C23	0.097 (3)	0.0531 (19)	0.077 (2)	0.0230 (18)	−0.009 (2)	0.0206 (16)
C24	0.0619 (18)	0.0491 (16)	0.0554 (17)	0.0160 (14)	0.0047 (14)	0.0161 (13)
C25	0.0547 (16)	0.0456 (15)	0.0424 (14)	0.0218 (13)	0.0105 (12)	0.0113 (12)
C26	0.0665 (19)	0.0612 (18)	0.0504 (17)	0.0098 (15)	0.0034 (14)	0.0164 (14)
C27	0.076 (2)	0.0629 (19)	0.060 (2)	0.0158 (16)	−0.0106 (17)	0.0071 (16)
C28	0.092 (3)	0.075 (2)	0.055 (2)	0.036 (2)	−0.0109 (18)	0.0027 (17)
C29	0.099 (3)	0.107 (3)	0.0460 (18)	0.037 (2)	0.0151 (18)	0.0272 (19)
C30	0.075 (2)	0.078 (2)	0.0485 (17)	0.0257 (17)	0.0118 (15)	0.0253 (16)
O4	0.0917 (19)	0.0811 (17)	0.137 (2)	0.0194 (15)	0.0375 (17)	0.0347 (16)
C31	0.086 (3)	0.097 (4)	0.252 (7)	0.021 (3)	0.026 (4)	0.080 (4)
C32	0.104 (3)	0.109 (4)	0.120 (4)	0.037 (3)	−0.004 (3)	0.015 (3)

### Geometric parameters (Å, °)

**Table:**

N1—C13	1.338 (3)	C15—H15A	0.9700
N1—C17	1.455 (3)	C15—H15B	0.9700
N1—C14	1.455 (3)	C16—C17	1.504 (4)
O1—C1	1.359 (3)	C16—H16A	0.9700
O1—C9	1.426 (3)	C16—H16B	0.9700
C1—C2	1.360 (3)	C17—H17A	0.9700
C1—C6	1.417 (4)	C17—H17B	0.9700
N2—C15	1.455 (3)	C18—C19	1.521 (3)
N2—C16	1.466 (3)	C18—C25	1.525 (3)
N2—C18	1.478 (3)	C18—H18A	0.9800
C2—C3	1.408 (3)	C19—C20	1.371 (4)
C2—H2A	0.9300	C19—C24	1.382 (4)
O2—C6	1.337 (3)	C20—C21	1.412 (5)
O2—C7	1.415 (4)	C20—H20A	0.9300
O3—C13	1.238 (3)	C21—C22	1.352 (6)
C3—C4	1.380 (3)	C21—H21A	0.9300
C3—C11	1.463 (4)	C22—C23	1.377 (6)
C4—C5	1.394 (4)	C22—H22A	0.9300
C4—H4A	0.9300	C23—C24	1.373 (4)
C5—C6	1.380 (4)	C23—H23A	0.9300
C5—H5A	0.9300	C24—H24A	0.9300
C7—C8	1.397 (5)	C25—C30	1.365 (4)
C7—H7A	0.9700	C25—C26	1.374 (4)
C7—H7B	0.9700	C26—C27	1.388 (4)
C8—H8A	0.9600	C26—H26A	0.9300
C8—H8B	0.9600	C27—C28	1.351 (5)
C8—H8C	0.9600	C27—H27A	0.9300
C9—C10	1.501 (4)	C28—C29	1.352 (5)
C9—H9A	0.9700	C28—H28A	0.9300
C9—H9B	0.9700	C29—C30	1.396 (5)
C10—H10A	0.9600	C29—H29A	0.9300
C10—H10B	0.9600	C30—H30A	0.9300
C10—H10C	0.9600	O4—C31	1.396 (5)
C11—C12	1.327 (3)	O4—H4B	0.8200
C11—H11A	0.9300	C31—C32	1.422 (6)
C12—C13	1.470 (3)	C31—H31A	0.9700
C12—H12A	0.9300	C31—H31B	0.9700
C14—C15	1.508 (4)	C32—H32A	0.9600
C14—H14A	0.9700	C32—H32B	0.9600
C14—H14B	0.9700	C32—H32C	0.9600
			
C13—N1—C17	120.8 (2)	C14—C15—H15B	109.5
C13—N1—C14	126.9 (2)	H15A—C15—H15B	108.0
C17—N1—C14	111.6 (2)	N2—C16—C17	112.5 (2)
C1—O1—C9	118.1 (2)	N2—C16—H16A	109.1
O1—C1—C2	125.1 (2)	C17—C16—H16A	109.1
O1—C1—C6	114.5 (2)	N2—C16—H16B	109.1
C2—C1—C6	120.4 (3)	C17—C16—H16B	109.1
C15—N2—C16	107.90 (19)	H16A—C16—H16B	107.8
C15—N2—C18	110.6 (2)	N1—C17—C16	111.5 (2)
C16—N2—C18	108.99 (19)	N1—C17—H17A	109.3
C1—C2—C3	121.2 (2)	C16—C17—H17A	109.3
C1—C2—H2A	119.4	N1—C17—H17B	109.3
C3—C2—H2A	119.4	C16—C17—H17B	109.3
C6—O2—C7	117.8 (3)	H17A—C17—H17B	108.0
C4—C3—C2	118.1 (2)	N2—C18—C19	110.71 (19)
C4—C3—C11	119.3 (2)	N2—C18—C25	111.4 (2)
C2—C3—C11	122.6 (2)	C19—C18—C25	111.6 (2)
C3—C4—C5	121.4 (3)	N2—C18—H18A	107.7
C3—C4—H4A	119.3	C19—C18—H18A	107.7
C5—C4—H4A	119.3	C25—C18—H18A	107.7
C6—C5—C4	120.2 (3)	C20—C19—C24	118.5 (3)
C6—C5—H5A	119.9	C20—C19—C18	119.2 (3)
C4—C5—H5A	119.9	C24—C19—C18	122.3 (2)
O2—C6—C5	125.4 (3)	C19—C20—C21	119.8 (3)
O2—C6—C1	115.8 (3)	C19—C20—H20A	120.1
C5—C6—C1	118.7 (3)	C21—C20—H20A	120.1
C8—C7—O2	113.1 (4)	C22—C21—C20	120.5 (4)
C8—C7—H7A	109.0	C22—C21—H21A	119.8
O2—C7—H7A	109.0	C20—C21—H21A	119.8
C8—C7—H7B	109.0	C21—C22—C23	120.0 (3)
O2—C7—H7B	109.0	C21—C22—H22A	120.0
H7A—C7—H7B	107.8	C23—C22—H22A	120.0
C7—C8—H8A	109.5	C24—C23—C22	119.6 (4)
C7—C8—H8B	109.5	C24—C23—H23A	120.2
H8A—C8—H8B	109.5	C22—C23—H23A	120.2
C7—C8—H8C	109.5	C23—C24—C19	121.6 (3)
H8A—C8—H8C	109.5	C23—C24—H24A	119.2
H8B—C8—H8C	109.5	C19—C24—H24A	119.2
O1—C9—C10	106.8 (3)	C30—C25—C26	118.0 (3)
O1—C9—H9A	110.4	C30—C25—C18	119.9 (3)
C10—C9—H9A	110.4	C26—C25—C18	122.1 (2)
O1—C9—H9B	110.4	C25—C26—C27	121.2 (3)
C10—C9—H9B	110.4	C25—C26—H26A	119.4
H9A—C9—H9B	108.6	C27—C26—H26A	119.4
C9—C10—H10A	109.5	C28—C27—C26	120.3 (3)
C9—C10—H10B	109.5	C28—C27—H27A	119.9
H10A—C10—H10B	109.5	C26—C27—H27A	119.9
C9—C10—H10C	109.5	C27—C28—C29	119.2 (3)
H10A—C10—H10C	109.5	C27—C28—H28A	120.4
H10B—C10—H10C	109.5	C29—C28—H28A	120.4
C12—C11—C3	127.6 (2)	C28—C29—C30	121.1 (3)
C12—C11—H11A	116.2	C28—C29—H29A	119.4
C3—C11—H11A	116.2	C30—C29—H29A	119.4
C11—C12—C13	121.2 (2)	C25—C30—C29	120.1 (3)
C11—C12—H12A	119.4	C25—C30—H30A	119.9
C13—C12—H12A	119.4	C29—C30—H30A	119.9
O3—C13—N1	121.3 (2)	C31—O4—H4B	109.5
O3—C13—C12	120.9 (2)	O4—C31—C32	118.1 (4)
N1—C13—C12	117.8 (2)	O4—C31—H31A	107.8
N1—C14—C15	109.7 (2)	C32—C31—H31A	107.8
N1—C14—H14A	109.7	O4—C31—H31B	107.8
C15—C14—H14A	109.7	C32—C31—H31B	107.8
N1—C14—H14B	109.7	H31A—C31—H31B	107.1
C15—C14—H14B	109.7	C31—C32—H32A	109.5
H14A—C14—H14B	108.2	C31—C32—H32B	109.5
N2—C15—C14	110.9 (2)	H32A—C32—H32B	109.5
N2—C15—H15A	109.5	C31—C32—H32C	109.5
C14—C15—H15A	109.5	H32A—C32—H32C	109.5
N2—C15—H15B	109.5	H32B—C32—H32C	109.5
			
C9—O1—C1—C2	−5.4 (4)	C15—N2—C16—C17	−56.8 (3)
C9—O1—C1—C6	172.3 (3)	C18—N2—C16—C17	−176.9 (2)
O1—C1—C2—C3	179.0 (2)	C13—N1—C17—C16	135.9 (3)
C6—C1—C2—C3	1.4 (4)	C14—N1—C17—C16	−52.7 (3)
C1—C2—C3—C4	1.2 (4)	N2—C16—C17—N1	53.7 (3)
C1—C2—C3—C11	−176.4 (2)	C15—N2—C18—C19	56.0 (3)
C2—C3—C4—C5	−1.7 (4)	C16—N2—C18—C19	174.4 (2)
C11—C3—C4—C5	176.0 (3)	C15—N2—C18—C25	−179.3 (2)
C3—C4—C5—C6	−0.5 (5)	C16—N2—C18—C25	−60.8 (3)
C7—O2—C6—C5	13.5 (5)	N2—C18—C19—C20	−134.7 (3)
C7—O2—C6—C1	−167.7 (3)	C25—C18—C19—C20	100.6 (3)
C4—C5—C6—O2	−178.2 (3)	N2—C18—C19—C24	45.2 (3)
C4—C5—C6—C1	3.0 (4)	C25—C18—C19—C24	−79.4 (3)
O1—C1—C6—O2	−0.3 (4)	C24—C19—C20—C21	0.1 (4)
C2—C1—C6—O2	177.6 (3)	C18—C19—C20—C21	−180.0 (3)
O1—C1—C6—C5	178.6 (3)	C19—C20—C21—C22	0.9 (5)
C2—C1—C6—C5	−3.5 (4)	C20—C21—C22—C23	−1.4 (6)
C6—O2—C7—C8	137.2 (4)	C21—C22—C23—C24	0.9 (6)
C1—O1—C9—C10	−172.3 (3)	C22—C23—C24—C19	0.1 (5)
C4—C3—C11—C12	−171.4 (3)	C20—C19—C24—C23	−0.6 (4)
C2—C3—C11—C12	6.2 (4)	C18—C19—C24—C23	179.5 (3)
C3—C11—C12—C13	175.4 (2)	N2—C18—C25—C30	121.6 (3)
C17—N1—C13—O3	−4.0 (4)	C19—C18—C25—C30	−114.1 (3)
C14—N1—C13—O3	−173.9 (3)	N2—C18—C25—C26	−59.7 (3)
C17—N1—C13—C12	175.1 (2)	C19—C18—C25—C26	64.6 (3)
C14—N1—C13—C12	5.2 (4)	C30—C25—C26—C27	−0.1 (4)
C11—C12—C13—O3	28.2 (4)	C18—C25—C26—C27	−178.9 (3)
C11—C12—C13—N1	−150.9 (3)	C25—C26—C27—C28	−0.1 (5)
C13—N1—C14—C15	−133.3 (3)	C26—C27—C28—C29	−0.5 (5)
C17—N1—C14—C15	56.0 (3)	C27—C28—C29—C30	1.2 (5)
C16—N2—C15—C14	60.3 (3)	C26—C25—C30—C29	0.9 (4)
C18—N2—C15—C14	179.4 (2)	C18—C25—C30—C29	179.6 (3)
N1—C14—C15—N2	−60.9 (3)	C28—C29—C30—C25	−1.4 (5)

### Hydrogen-bond geometry (Å, °)

**Table:**

*D*—H···*A*	*D*—H	H···*A*	*D*···*A*	*D*—H···*A*
O4—H4B···O3	0.82	1.94	2.765 (3)	177
C11—H11A···O3	0.93	2.53	2.845 (3)	100
C17—H17A···O3	0.97	2.33	2.734 (4)	104
